# You got me (un)hooked: Repair of DNA crosslinks in bacteria

**DOI:** 10.1590/1678-4685-GMB-2026-0025

**Published:** 2026-10-26

**Authors:** Henrique Kustor, Valdir Cristovão Barth, Rodrigo S. Galhardo

**Affiliations:** 1 Universidade de São Paulo, Instituto de Ciências Biomédicas, Departamento de Microbiologia, São Paulo, SP, Brazil.

**Keywords:** DNA Repair, Interstrand Crosslinks, Intrastrand Crosslinks, DNA Monoadducts, Bacteria

## Abstract

DNA crosslinks are cytotoxic lesions caused by a variety of DNA reacting compounds. Some bacterial competitors and environmental organisms evolved the ability to synthesize and secrete alkylating molecules that bind to DNA, impairing replication and transcription and ultimately resulting in death of the target cells. Notwithstanding, environmental exposure, spontaneous chemical reactions and byproducts of the physiological metabolism can interfere with DNA, leading to toxic crosslinking damage. Hence, bacteria evolved repair systems capable of removing lesions and allowing cells to thrive even in harsh conditions. Base Excision Repair, Nucleotide Excision Repair, Homologous Recombination and Translesion Synthesis all combine to solve DNA monoadducts, Intrastrand, Interstrand, and DNA-Peptide Crosslinks. In this review, we address major stressors to which bacteria are exposed, their DNA-damaging capacities and how the prokaryotic cell responds.

## DNA crosslinks: Agents and damages

Bacterial cells spanning diverse ecological niches and habitats are under constant genotoxic distress. The host immune response, microbiome competitors, adaptive resistance mechanisms, pollutants, naturally occurring aggressors and the cell metabolism itself all pose as DNA damage sources (Wozniak and Simmons, 2022). Altogether, these elements induce lesions which come in very different colors and shapes. To cope with those, evolution has selected multiple repair and tolerance pathways in bacteria. While some damages may be resolved by single enzymes, others require the recruitment of two or more systems combined (Noll et al., 2006). That is the case for interstrand crosslinks (ICLs), a harsh type of lesion that hooks opposing DNA strands together through covalent bonds, requiring an integrated, multiple-step repair pathway. Yet, different enzyme sets may be required according to the crosslinking molecule, even for seemingly similar lesions (Berardini et al., 1997). In this review, we assess the diversity of DNA crosslinking agents present in bacteria-colonized environments and how bacterial cells deal with that genotoxic stress.

DNA crosslinks are a result of the reaction between alkylating agents and DNA, forming stable covalent bonds (Lawley, 1966). Crosslinking agents can be classified as monofunctional or bifunctional. The monofunctional agents are those that possess a single reactive group, therefore binding on a single site of the DNA to form a monoadduct. On the other hand, bifunctional agents carry two or more reactive groups, being able to bind two different residues either from the same strand, forming an intrastrand crosslink, or from different strands, leading to an ICL. Proteins can also be crosslinked to DNA, forming the so-called DNA-Protein Crosslinks (DPCs) (Tretyakova et al., 2015). Overall, those lesions may or may not unwind, distort and separate DNA strands, and flip, tilt or modify bases, interfering with replication and transcription, thus showing cytotoxic and mutagenic effects.

## Collateral effect: Endogenous crosslinking agents

Another DNA damage source faced by bacteria lies in its own metabolic byproducts. Reactive oxygen and nitrogen species (ROS and RNS) are constantly generated inside the cell even under normal conditions. These reactive species interact with most cell components, DNA included. Oxidative stress may be the major cause of DNA damage under physiological conditions, also playing a role in DNA crosslinks (Geiduschek, 1961). Oxidative damage alters base identity (Cadet et al., 2017), for example, guanine becomes 8-oxoguanine (8-oxoG) (Figure 1 A ). Damaged base processing through base excision repair results in apurinic/apyrimidinic (AP) site intermediates (Wozniak and Simmons, 2022), which also occur spontaneously through hydrolysis of either normal or oxidized bases (Gates, 2009). ROS can also react with the deoxyribose, simultaneously provoking a single strand break (SSB) and an oxidized 5’ AP site (5’-(2-Phosphoryl-1,4-dioxobutane), or DOB) (Chin et al., 1987) (Figure 1 B ). The deoxyribose from AP DNA may undergo a spontaneous opening of the sugar ring, exposing an aldehyde moiety, which is then capable of reacting with either adenine or guanine of the opposite strand, forming an ICL (Dutta et al., 2007; Guan and Greenberg, 2009).

**Figure 1 -:**
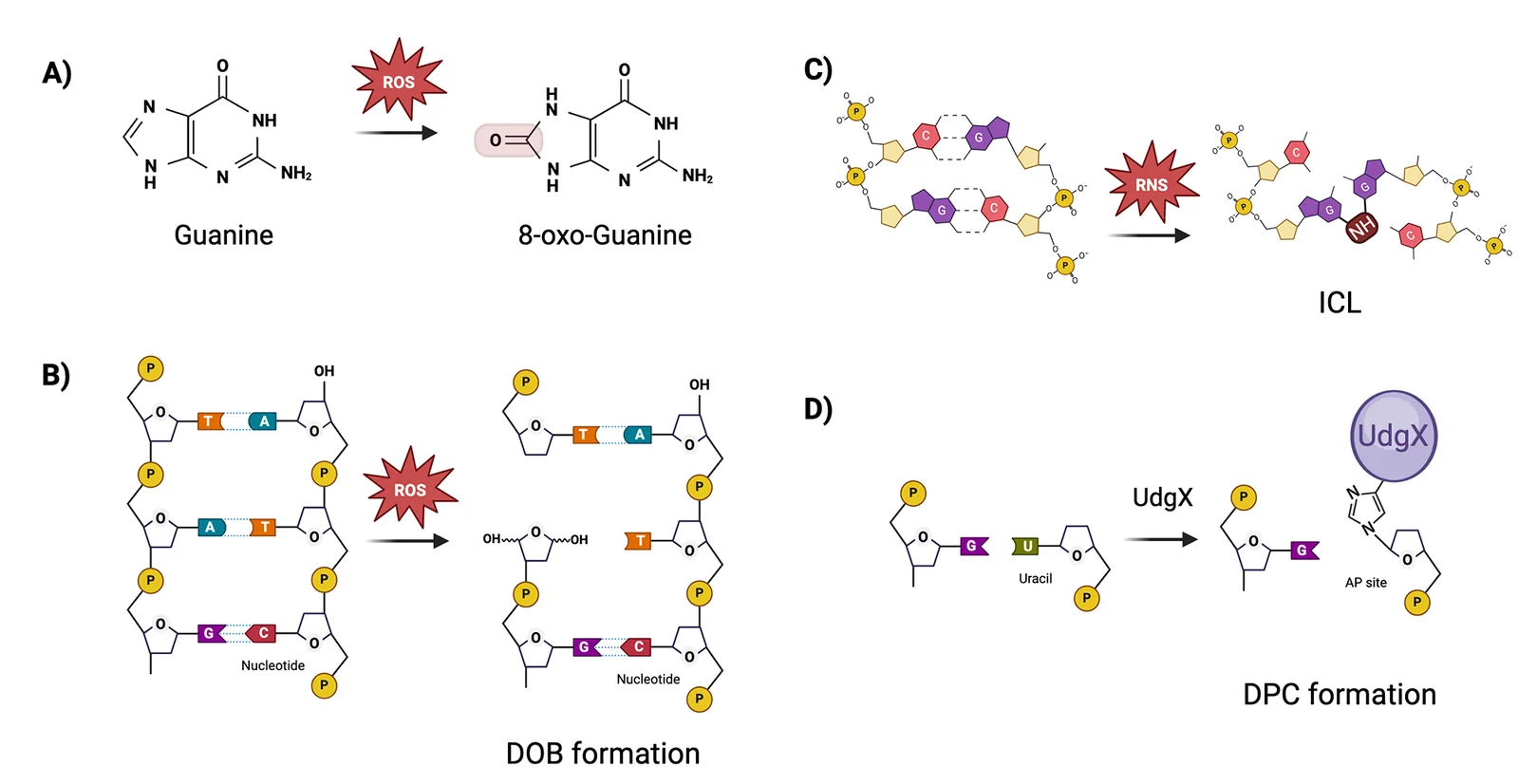
DNA damage caused by endogenous agents, such as Reactive Oxygen Species (ROS), Reactive Nitrogen Species (RNS) and DNA-Protein crosslinks. A) The oxidation of bases such as Guanine leads to the formation of 8-oxo-Guanine. B) ROS can react with the deoxyribose generating a 5’-(2-Phosphoryl-1,4-dioxobutane), or DOB, which is an abasic sugar residue created simultaneously with a single strand break. C) RNS such as •NO or HNO_2_ can generate an ICL directly connecting two Guanines via a shared imino group in the opposite strands of DNA, leading to severe strand distortion. D) The enzyme UdgX covalently attaches via a Histidine residue to the C1 carbon of the sugar in the resulting abasic site following the excision of an Uracil.

Nitrifying bacteria also produce nitrous acid (HNO_2_) as a subproduct of nitrite metabolism. Both HNO_2_ and its derivative, nitric oxide (•NO), are DNA damaging agents, reacting with guanine residues and leading to their deamination (Caulfield et al., 1998). The deaminated guanines at CpG sites may then react with another guanine from the opposing strand (Figure 1 C ), resulting in an ICL which severely tilts affected bases and distorts DNA (Edfeldt, 2004). ROS and RNS can also induce the occurrence of both DNA and protein radicals that cause DPCs, as does ionizing radiation (Kojima and Machida, 2020).

Furthermore, DNA metabolism itself can lead to DPC formation. For example, the bacterial UdgX family of uracil-DNA glycosylases - which remove uracil from DNA as part of the base excision repair - covalently bind to the AP site generated upon uracil excision (Figure 1 D ) (Ahn et al., 2019). Incomplete processing by topoisomerases may also result in DPCs, as these enzymes break, unwind and ligate the DNA double strand to relieve its tension, generating simultaneously DNA end-protein crosslinks and double strand break (DSB) intermediates (Wojtaszek and Williams, 2024). The correct functioning of this system is so important that it has been targeted for antibiotic development, and compounds that trap topoisomerases in its toxic intermediate state are currently in use to fight bacterial infections (Rodrigues and Silva, 2025). Another endogenous protein implicated in DPC formation is *E. coli* YedK, a SOS Response-Associated Peptidase (SRAP) domain-containing enzyme which reversibly crosslinks itself to AP sites (Mohni et al., 2019; Paulin et al., 2022), including 3’ ends generated by DNA lyases, possibly with a protective function to avoid further damage or mutations, as YedK is able to catalyze the reversal of its ligation to DNA *in vitro*.

## Biological warfare: Competitors and host-derived compounds

Multiple bacterial and plant species have developed the ability to synthesize and secrete DNA-damaging compounds to eliminate competitors and pathogens. Some of those rely on a crosslinking mode of action that kills exposed bacterial cells. 

Bacteria from the *Streptomyces* genus produce more than one DNA crosslinking agent. Mitomycin C (MMC) is an extensively studied molecule produced by *S. caespitosus* (Hata et al., 1956)*,* which has been used as a chemotherapeutic drug to eliminate tumor cells. MMC (Figure 2 A ) is a bifunctional alkylating agent that reacts with the N2 position of guanines, specifically at 5’-CG-3’ sequences in DNA minor grooves, to form ICLs (Tomasz, 1995). This base preference is explained by its lower oxidation potential, making guanine a recurrent target for reactive species (Jena and Mishra, 2012). While MMC is originally inert, it undergoes a chemical or enzymatic reduction step to become reactive. It binds to DNA initially as a monoadduct, then a series of other aqueous reactions are necessary to crosslink to the opposing strand (Rajski and Williams, 1998). Apart from ICLs, which account for a minority of MMC-induced lesions, monoadducts are actually the most frequent product coming from ICL intermediates or the alkylation of adenines at their N6 positions (Tomasz, 1995). Furthermore, these ICLs constitute only a minor distortion of the DNA structure (Tomasz *et al*., 1987), challenging lesion recognition. Another compound produced by a *Streptomyces* sp. is azinomycin B (AZB), isolated from *S. sahachiroi*. AZB (Figure 2 B) is a bifunctional alkylating agent that reacts with purines at the N7 position (Armstrong et al., 1992), initially causing monoadducts. Even though AZB reacts with adenine in 5’-RNY-3’ sites, there is a preference for guanines, especially at 5’-GGC-3’ sites in the major groove, crosslinking to the opposite guanine and resulting in ICLs (Coleman et al., 2002).

**Figure 2 -:**
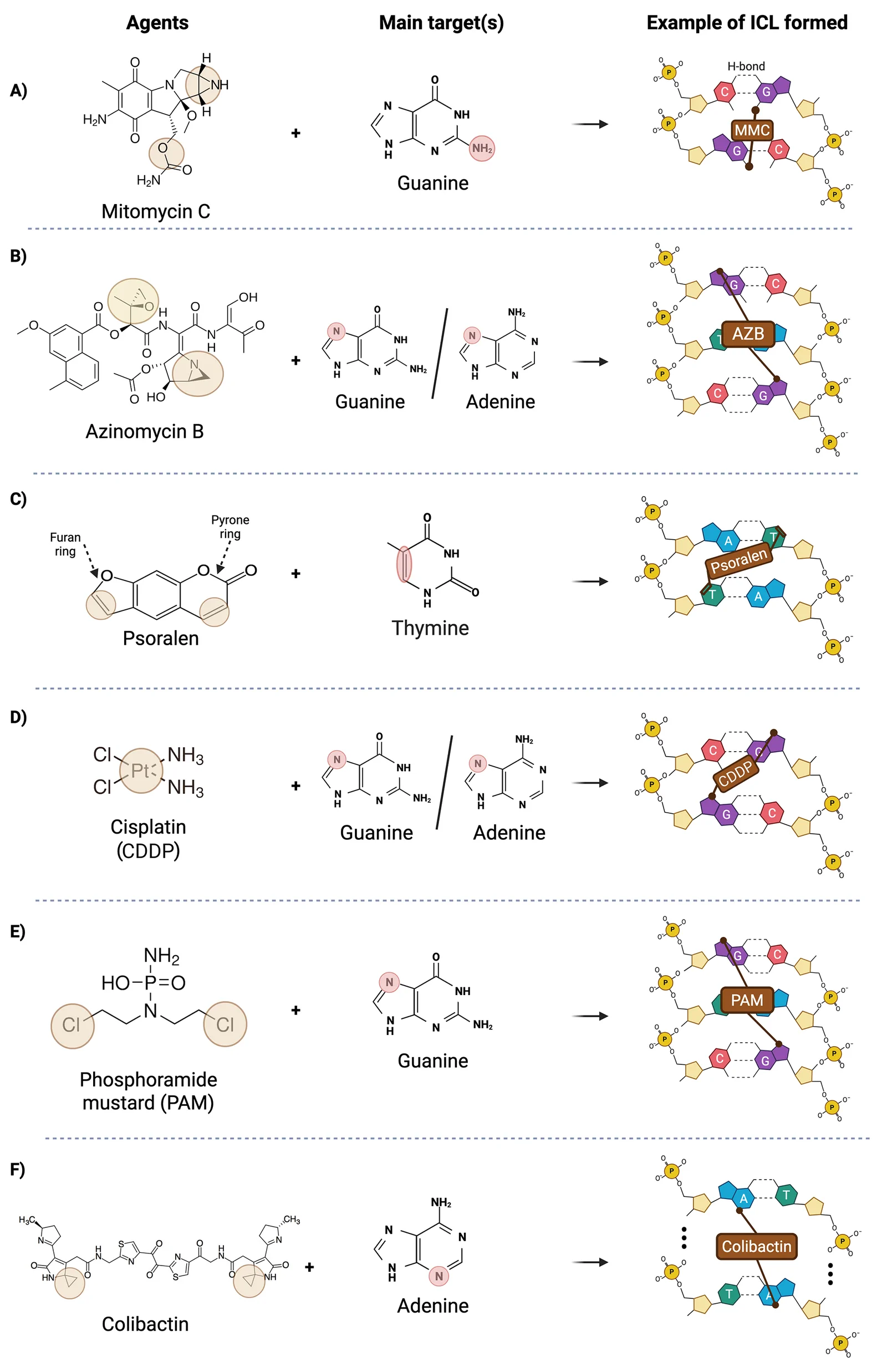
Alkylating agents that cause ICLs, their target nucleotide base(s) and a representative crosslink formation. Reactive groups of the agents are circled in yellow, while their target group in the nitrogen base is circled red. A) Mitomycin C crosslinks Guanines at N2 amino groups. B) Azinomycin B can generate crosslinks in Adenine or Guanine at N7 of a 5’-Pur-N-Pyr-3’ site. C) Psoralen upon photoactivation targets pyrimidine bases (especially Thymine) undergoing a cycloaddition reaction to form a covalent monoadduct that can further react with the complementary strand to form an ICL at ApT sites. D) Cisplatin can react with the N7 of Guanine (or Adenine) forming intra-strand cross-links (not shown) or, less frequently, an ICL. E) Phosphoramide mustard can crosslink two opposing Guanines at N7 on 5’-GNC-3’ sites. F) Colibactin can form ICL by reacting with N3 of Adenines on opposite strands, preferentially within a 5′-WAWWTW-3′ motif, targeting two Adenines with a 3-4 bp distance in the DNA minor groove.

The Enterobacteriaceae family is another bacterial clade known to synthesize DNA-crosslinking molecules. Species from this family, including the clinically relevant *Escherichia coli* and *Klebsiella pneumoniae* - responsible for a vast burden of global nosocomial infections, notably urinary tract infections, bacteremia, and pneumonia - can harbor the polyketide synthase (*pks*) genomic island encoding enzymes that produce the inactive prodrug precolibactin (Nougayrède et al., 2006; Putze et al., 2009), which suffers deacetylation to become the crosslinking colibactin (Figure 2 F ) (Bossuet-Greif et al., 2018). This agent presents a bis-electrophilic DNA crosslinking mechanism, leading to ICL formation between adenines in the DNA minor groove, with a 5’-WAWWTW-3’ sequence preference (Wilson et al., 2017; Xue et al., 2019; Carlson et al., 2025). Nonetheless, colibactin-DNA ICLs are rather unstable, leading to depurination of the crosslinked adenines followed by 3’ phosphate elimination, which accounts for SSBs and subsequent DSBs (Xue *et al*., 2020).

Along with bacteria, plants also synthesize a well-known group of crosslinking agents, the psoralens (Figure 2 C ). These compounds present furan and pyrone rings on opposite sides of the molecule, both capable of alkylating DNA upon activation by long wavelength ultraviolet (UV) light (Parrish et al., 1974). Psoralens and UV-A (PUVA) are combined for therapeutic purposes, serving as treatment for skin conditions such as psoriasis, vitiligo and cutaneous T-cell lymphoma (Noll et al., 2006). Psoralens preferentially react with thymine at 5’-TA-3’ sites (Cimino et al., 1985; Gia et al., 1992), and its capacity to induce interstrand crosslinks (ICLs) depends entirely on the molecule’s initial binding orientation. While a pyrone-side monoadduct cannot absorb a second photon and remains an unreactive monoadduct, a furan-side monoadduct can be further photoactivated to form a crosslink with the opposing DNA strand. In a successful multi-photon reaction, psoralen forms two cyclobutane rings: one between its furan ring and a thymine on the first strand, and a second between its pyrone ring and a complementary thymine on the opposite strand. PUVA induces these ICLs with high efficiency, accounting for 40% to 90% of total lesions depending on the use of methoxypsoralen or trimethylpsoralen, respectively (Dronkert and Kanaar, 2001; Huang *et al*., 2013). These ICLs have a mild unwinding effect on the double helix, but do not induce significant DNA axial bending (Rycenga and Long, 2018). Because psoralen-DNA ICLs can be cleanly generated via controlled UVA irradiation, they are widely utilized as highly tractable models for studying DNA repair mechanisms *in vitro* (Noll *et al*., 2006).

## Chemotherapeutic drugs and other environmental sources of damage

In some cases, bacteria may also be exposed to host-directed agents that cause DNA crosslinks. Platinum-based compounds and nitrogen mustards have been widely used in cancer therapy, apart from the previously mentioned MMC and PUVA. These drugs kill replicating tumor cells through DNA damage, but can also affect healthy cells and bacteria whether within the host or in the environment (Lehouritis et al., 2015; Castellano-Hinojosa et al., 2023). For that purpose, some chemotherapeutic agents have been explored as alternatives to fight multi-drug resistant bacterial infections (Chowdhury et al., 2016). The most common platinum compound is cis-diamminedichloroplatinum (CDDP, or simply cisplatin), which loses its chloride through an aquation reaction inside the cell, exposing the platinum ion to react mostly with guanine residues in DNA major groove and forming intrastrand crosslinks at 5’-GG-3’, 5’-GNG-3’ or 5’-AG-3’ sequences (Fichtinger-Schepman et al., 1985). Although it occurs rarely, cisplatin can also form ICLs that cause significant distortion of the double helix and cytosine flipping at 5’-GC-3’ sites (Figure 2D) (Malinge et al., 1999).

On the other hand, cyclophosphamide is a widely used bifunctional agent, but it has to be metabolized into phosphoramide mustard (PAM) in the liver and inside cells to become reactive (Sladek, 1988). Nonetheless, active compounds may be excreted through the urinary tract (Joqueviel et al., 1998). PAM and other nitrogen mustards also react with guanines to initially form a monoadduct, later to react with water and stand as it is, or with another guanine in 5’-GNC-3’ sites, generating ICLs that bend and unwind the DNA helix (Figure 2 E ) (Rink and Hopkins, 1995; Dong et al., 1995).

Finally, human activity results in environmental release of pollutants, such as formaldehydes and polycyclic aromatic hydrocarbons from incomplete combustion in engines (Kojima and Machida, 2020). These molecules cause different DNA crosslinks, but mainly DPCs between lysine residues and guanine (Lu et al., 2010).

## Base excision repair

The canonical base excision repair (BER) pathway plays only a minor role in the repair of DNA crosslinks itself, but prevents single damaged residues from acting as intermediates for crosslinks. Given that this topic was comprehensively reviewed by Wozniak and Simmons (2022), it will be briefly summarized in this section (Figure 3). BER is usually initiated by highly damage-specific DNA glycosylases. For instance, MutM (or Fpg) primarily targets oxidative DNA lesions, including 8-oxoG and various modified purines, whereas UDGs are specific for the removal of uracil. These enzymes operate by breaking the N-glycosidic bond between the deoxyribose and the altered base, resulting in an AP site. Whether generated as an intermediate of DNA repair or directly by damaging agents, the processing of these lesions may be the most important BER activity for crosslink prevention and can be carried out by different pathways depending on which enzyme acts next.

**Figure 3 -:**
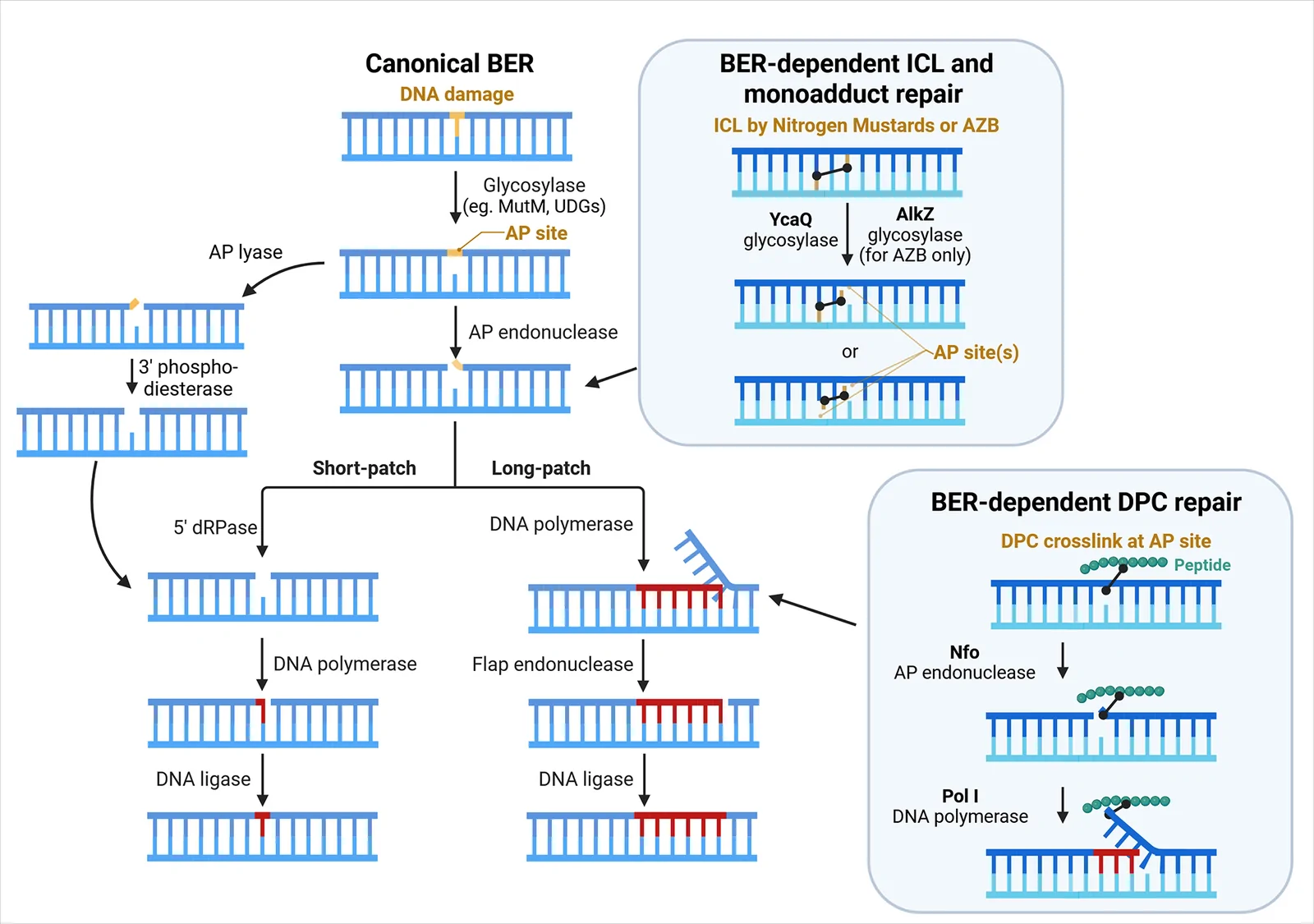
Models of the canonical Base Excision Repair (BER) and BER-dependent repair pathways of ICLs and DPCs. The canonical BER pathway initiates when a glycosylase removes a damaged base to create an Apurinic/Apyrimidinic (AP) site, which is subsequently processed by either an AP lyase or an AP endonuclease. After AP lyase incision 3’ of the abasic site, a 3’ phosphodiesterase removes the 3’ intermediate, leaving a 3’-OH necessary for repair. On the other hand, after AP endonuclease 5’ incision, the pathway branches into short-patch BER, in which dRPase removes the 5’ intermediate, DNA polymerase completes a single nucleotide gap and a ligase seals the nick, or long-patch BER, where DNA polymerase performs strand displacement to create a flap which is excised by a flap endonuclease. ICLs and monoadducts from nitrogen mustards or azinomycin B (AZB) are unhooked by YcaQ or AlkZ glycosylases into AP site intermediates that enter the canonical pathway. Similarly, DNA-Protein Crosslinks (DPCs) at AP sites are cleaved by Nfo AP endonuclease, followed by DNA Pol I-mediated strand displacement and repair via the long-patch sub-pathway.

Some DNA glycosylases are characterized as bifunctional (e.g. Nth), possessing an AP lyase activity that breaks the phosphodiester bond at the 3’ side of the generated AP site. This yields a 5’-phosphate and a 3’-unsaturated aldehyde end, which is later removed from DNA by a 3’ phosphodiesterase, resulting in a single nucleotide gap - a process termed short-patch BER. Alternatively, DNA backbone cleavage can be made by AP endonucleases 5’ of the AP site, generating 5’-deoxyribose phosphate (dRP) and 3’-OH ends and leading to two possible resolutions: in short-patch BER, a deoxyribophosphodiesterase (dRPase) excises the 5’ dRP group, leaving a single-nucleotide gap that is subsequently filled by a DNA polymerase. Alternatively, during long-patch BER, strand-displacement DNA synthesis occurs, displacing the affected 5’ strand into a single-stranded DNA flap that is later cleaved by a flap endonuclease. Both pathways ultimately converge at the final step, where a DNA ligase seals the remaining nick to complete the repair process. 

## Base excision repair of crosslinks

Apart from AP site repair and the removal of lesions that may potentially lead to ICL formation, some bacterial species possess specialized DNA glycosylases that initiate monoadduct and ICL repair, but are specific to certain damaging agents (Figure 3, top-right box). The characterized enzymes with such activity are members of the InterPro database DNA-glycosylase AlkZ-like family (IPR009351) (Blum et al., 2025), which is distributed across diverse bacterial phyla, including a significant number of species from Actinomycetota, Bacillota, Pseudomonadota, Bacteroidota and Deinococcota. 

The YcaQ glycosylase, characterized in *E. coli* (Bradley et al., 2020), is able to remove nitrogen mustard (NM) and AZB monoadducts, or unhook those same ICLs, leaving an AP site on one strand and the crosslinked bases still bound to the other. Furthermore, a YcaQ homologue from *Acinetobacter baumannii*, AlkX, was recently shown to process NM ICLs (Cai et al., 2026), and is believed to play a role in the pathogenesis of this clinically relevant bacterium, affecting lung colonization and tissue invasion on pneumonia, as well as acidic pH resistance (Kunkle et al., 2024). Another BER example is AlkZ from *Streptomyces* spp., an enzyme encoded in the AZB biosynthetic cluster, and highly specialized in removing monoadducts and unhooking ICLs caused by that agent (Wang et al., 2016; Mullins et al., 2017). Both YcaQ and AlkZ were shown to excise the crosslinked bases from both affected strands *in vitro*, generating opposing AP sites prone to cleavage by the Nfo AP endonuclease (*E. coli* Endonuclease IV), resulting in double-strand breaks (Bradley *et al*., 2020).

The AP endonuclease Nfo was also shown to efficiently cleave the phosphodiester bond at small AP-peptide crosslinks (Bryan et al., 2022), or following proteolysis of a larger adduct (Yudkina et al., 2023), resulting in a free 3’-OH end and a 5’ side crosslinked peptide, which is a substrate for the Pol I strand displacement synthesis (Figure 3, bottom-right box). This activity results in a 5’ flap DNA containing the DPC, that is cleaved by Pol I flap endonuclease activity while the repair is completed by DNA ligase (Bryan *et al*., 2022).

## Nucleotide excision repair

The nucleotide excision repair (NER) is the most versatile damage repair system in bacteria (reviewed in Wozniak and Simmons, 2022), acting on a variety of bulky lesions, including monoadducts, intrastrand crosslinks and DPCs, while also having a major role in ICL repair. This pathway (Figure 4 A ) starts with UvrA dimerization and ATP binding in the proximal and distal enzyme sites. UvrA_2_ scans the DNA, and once damage is recognized, ATP from the proximal site is hydrolyzed. Next, one UvrB unit binds to each UvrA, ATP from the distal site is hydrolyzed and UvrA_2_ is released. UvrB possesses 5’-3’ helicase activity, and the two units will unwind DNA in convergent directions. Once a UvrB monomer finds and binds to the lesion, the other monomer is released and UvrC endonuclease is recruited, promoting simultaneous single-strand incisions upstream and downstream of the damaged site. Alternatively, in certain cases, the Cho endonuclease may cooperate with UvrC by making the 3’ incision (Moolenaar et al., 2002). The enzymes are released from DNA and UvrD helicase removes the nicked fragment, so DNA polymerase can fill the gap and DNA ligase seals it (Wozniak and Simmons, 2022).

**Figure 4 -:**
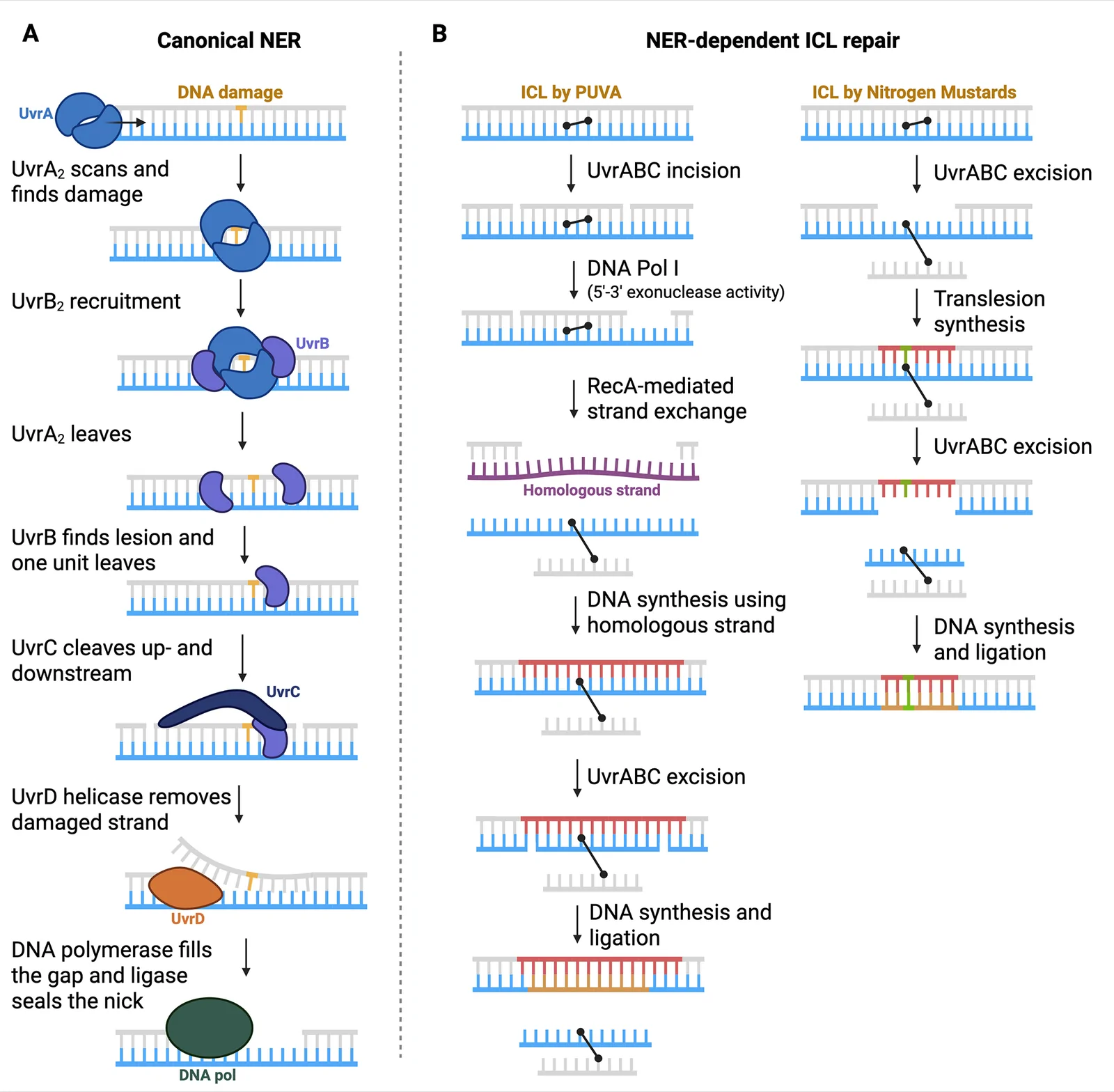
Models of canonical Nucleotide Excision Repair (NER) and two examples of NER-dependent Interstrand Crosslink (ICL) repair pathways in bacteria. A) Canonical NER Pathway: The UvrA_2_ homodimer continuously scans the DNA double helix to detect DNA damage. Once the lesion is identified, UvrB is recruited to form a UvrA_2_B_2_ complex. The UvrA_2_ dimer dissociates, allowing UvrB to reach the damage site while the second UvrB subunit leaves. UvrC is recruited and catalyzes incisions both upstream and downstream of the lesion. The UvrD helicase displaces the damaged oligonucleotide strand. Finally, DNA polymerase and DNA ligase fill and seal the gap. B) NER-dependent ICL Repair: PUVA-induced ICLs repair (left) uses UvrABC to detect and cleave up- and downstream of the damage. The 5’-3’ exonuclease activity of DNA Polymerase I creates a single stranded region. RecA then mediates strand exchange with an undamaged homologous strand (purple), which serves as a template for DNA synthesis to bypass the lesion. A second UvrABC excision event removes the remaining crosslinked adduct, followed by final DNA synthesis and ligation to restore the double helix. The Nitrogen Mustard-induced ICLs repair (right) utilizes an initial UvrABC excision to unhook the crosslink. Translesion synthesis polymerases synthesize DNA directly past the obstructing lesion (indicated by the red nucleotide). A second round of UvrABC excision removes the crosslinked adduct on the opposite strand, and the repair is completed by DNA polymerase and DNA ligase.

## NER-associated pathways

Dealing with DNA damage may require the activation of the bacterial SOS Response (reviewed in Lima-Noronha et al., 2022). Bulky lesions, including those caused by alkylating agents, may result in the exposure of ssDNA tracts, for instance due to replication fork stalling or as repair intermediates. Once that happens, RecA monomers bind to the ssDNA to form nucleoprotein filaments and turn into their active state (RecA*), which induces the self-cleavage of the LexA transcriptional repressor. This leads to the expression of the SOS Response genes, encoding different sets of DNA repair and damage tolerance proteins across the Bacteria domain. As will be further addressed, completing the repair of ICLs is a multi-pathway task. Along with NER, both homologous recombination (HR) and translesion synthesis (TLS) play an important role. Hence, different components of the crosslink repair machinery may be part of the LexA-regulon.

Beyond its regulatory role, RecA also takes a key part in HR by promoting the ssDNA end invasion of a homologous dsDNA, a process also referred to as synapsis. HR is a complex system involved in various cellular processes and was reviewed by Rocha et al. (2005) and more recently by del Val et al. (2019) and Bianco (2025), therefore it will only be briefly summarized here. As evidenced earlier, BER and NER introduce single-stranded nicks that are processed into gaps requiring DNA resynthesis. However, if the complementary template strand is also damaged (e.g., by an unhooked ICL) and a DNA polymerase cannot fill the gap, the HR pathway may be recruited to bypass the block via template switching. Moreover, structurally analogous single-stranded gaps arise when replication forks stall, leaving the newly synthesized nascent strand truncated and exposing a vulnerable template tract. To protect ssDNA from further damage, single-stranded DNA binding proteins (SSBPs) cover the exposed tract. RecFOR proteins then act to displace SSBPs, allowing RecA nucleofilament assembly and HR-mediated strand exchange. 

When replication forks encounter blocking lesions such as crosslinks, replisome collapse may occur and DNA repair enzymes can break phosphodiester bonds. If replication restarts before DNA ligation and replicative polymerase reaches the template nick, it undergoes replication runoff, converting the single-strand break into a one-sided double-strand break. The RecBCD/AddAB complex is then recruited to promote DNA end resection through its combined helicase/nuclease activity, generating a RecA nucleoprotein filament and leading to HR synapsis. To that end, the Structural Maintenance of Chromosomes (SMC) family protein RecN plays an essential role by acting as a cohesin that holds together sister chromatids and stimulates strand exchange. Nonetheless, RecN function is critical to MMC-induced damage repair (Noda et al., 2023). Finally, those strand exchange events result in the formation of Holliday junctions, which are later reversed by the activity of RuvABC/RuvAB RecU resolvases or RecG helicase. 

TLS is a damage tolerance alternative rather than a proper repair pathway, therefore it will not be deeply addressed in this work, but the interested reader is encouraged to review this subject in the work published by Fuchs and Fujii (2013). Intact ICLs impede strand separation and thus cannot be bypassed by TLS. Conversely, when replication forks stall at damaged sites such as monoadducts, intrastrand crosslinks and unhooked ICLs, specialized error-prone polymerases can be recruited to bypass the lesion and restart DNA replication, sometimes at the expense of fidelity. Even though distinct polymerases are scattered across bacterial species and oftentimes co-occur, the literature on crosslinks TLS in bacteria is scarce and centered on *E. coli*. In this organism, cisplatin-induced mutagenesis was shown to be dependent on the Y-family Umu'_2_DC polymerase (Pol V) (Fram et al., 1985). Also, the Y-family DinB (Pol IV) and the B-family Pol II are able to bypass unhooked aldehyde- and NM-induced ICLs, respectively, but the contrary does not occur (Berardini et al., 1999; Kumari et al., 2008).

## Canonical interstrand crosslink repair

If a single strand is affected, NER is sufficient to fulfill repair without requiring other enzyme sets. However, ICLs are lesions that hook both DNA strands together. The classical models describe NER as a strictly necessary component in ICL repair (Noll et al., 2006), although this requirement has recently been challenged (Cole et al., 2018). In fact, more recent studies suggest the existence of NER-independent systems, for instance the MmcB nuclease acting in concert with translesion synthesis in *Caulobacter crescentus* (Lopes-Kulishev et al., 2015). 

Tackling ICLs requires a combination of multiple molecular machineries in an agent-dependent manner. The best described models include PUVA or nitrogen mustard lesions (Figure 4 B ), with NER respectively acting in concert with HR or TLS (Van Houten et al., 1986; Sladek et al., 1989; Berardini et al., 1999). Either way, repair initiates with NER enzymes unhooking the crosslink and generating a single-stranded gap on one strand, while the other strand remains with a crosslinked DNA fragment. The next step is filling the gap, at which point different pathways may operate, for example, homologous recombination may provide an undamaged template (Figure 4 B ). After one strand is corrected, NER is once again recruited to solve the intermediate. 

Regarding PUVA ICL, Cho endonuclease was shown to participate in the NER-mediated repair (Perera et al., 2016). After the furan-adduct unhooking, DNA Polymerase I initiates a 5’ exonucleolytic cleavage from the UvrC/Cho nick, downstream of the lesion (Sladek et al., 1989). Then, RecA mediates the synapsis of the upstream 3’OH end with homologous DNA, introducing an undamaged, complementary DNA strand to the replication machinery (Van Houten et al., 1986). Once the nicked strand is resynthesized, Holliday junctions are resolved and the initial gap is sealed. As for nitrogen mustard ICLs, after the unhooking, TLS Pol II can synthesize DNA through the first NER intermediate and fill the gap (Berardini et al., 1999). After one strand is successfully repaired by either pathway, canonical NER takes place and completely removes the crosslink by nicking the opposing strand (Figure 4 B ).

## Non-canonical repair of DNA crosslinks

BER and NER are ubiquitous among bacteria, but for some species the repertoire of repair enzymes that solve DNA crosslinks goes beyond those pathways, as other DNA metabolism proteins have been shown to play a role in survival against crosslinking agents and damage reversal. Moreover, as mentioned earlier, the classical model for ICL repair through NER-HR and NER-TLS pathways was put into question by Cole and colleagues (Cole et al., 2018). While the authors only examined *E. coli*, their results suggest that neither NER, HR nor TLS efficiently repair or promote tolerance against PUVA-induced ICLs, but rather aids cell survival only against monoadducts.

One NER-independent system is the MrfAB pathway described in *Bacillus subtilis* and also found among some species from Bacillota and Pseudomonadota phyla (Burby and Simmons, 2019; Manthei et al., 2024). The MMC resistance factors A and B consist of a helicase and a 3’-5’ DNA exonuclease, respectively, which act in concert to repair MMC-induced monoadducts and intrastrand crosslinks, but not ICLs. Yet, at least one other factor of this pathway is still unknown.

Another interesting non-canonical bacterial ICL repair mechanism is the one involving a FAN1 homologue from *Pseudomonas aeruginosa*. FAN1 (FancI/FancD2-associated nuclease 1) is a eukaryotic enzyme present in a variety of organisms, including humans, fungi and plants, while the InterPro Fanconi-associated nuclease 1-like family (IPR033315) also has representatives among the Gammaproteobacteria, Betaproteobacteria and Cytophagia prokaryotic classes. The bacterial protein was shown to act upon 5’ DNA flaps followed by ICLs, promoting both endonucleolytic excision of the flap and then unhooking of the crosslink through trinucleotide excisions (Gwon et al., 2014; Jin et al., 2018). It is tempting to speculate that NER could be involved in intermediate resolution and completing the ICL repair, though the exact pathway has not yet been determined.

Furthermore, *E. coli* UvrB was previously believed to repair ICLs in a NER-independent fashion (Lage et al., 2010), but subsequent studies have refuted that hypothesis (Perera et al., 2016). Likewise, *C. crescentus* MmcB nuclease was shown to protect bacteria from MMC cytotoxic effect independently of UvrA (Lopes-Kulishev et al., 2015). This enzyme is the founding member of the DNA repair protein MmcB-like (IPR009394) InterPro family, which is distributed almost exclusively among Alphaproteobacteria. MmcB is believed to unhook MMC ICL by nicking one strand both up and downstream of the lesion, although no enzymatic activity was demonstrated supporting this hypothesis. Such activity would create a substrate for the TLS polymerase ImuC, similarly to the NER-TLS pathway, and resulting in mutagenesis-prone repair. 

Another example pending full characterization is the repair mediated by *Staphylococcus aureus* DinG helicase. This enzyme possesses an N-terminal 3’-5’ exonuclease domain that functions in concert with the helicase activity to prevent MMC and formaldehyde cytotoxic effects (Gao et al., 2025). Members of the 3’-5’ exonuclease DinG family (IPR006310) are limited to the Bacilli class. There is also a damage-induced operon from *Mycolicibacterium smegmatis* encoding two enzymes with protective function against crosslinking agents, the Lhr helicase and Nei2 glycosylase. While Lhr-deficient strains show increased sensitivity to MMC, cisplatin and PUVA, Nei2 is only effective against the latter, though the complete pathway and mechanisms remain unknown (Warren et al., 2023; Warren and Shuman, 2024). 

## DNA-peptide crosslink repair

As a consequence of molecular size, repairing DPCs is quite challenging for the bacterial cell. Although a single strand is affected, NER is unable to remove DNA fragments crosslinked to large proteins. It has been demonstrated that eukaryotic cells rely on proteolysis by dedicated enzymes to generate intermediates which DNA repair pathways are able to assess (Vaz et al., 2017), but there is no clear evidence at this time for a similar system in bacteria. In fact, *E. coli* ATP-dependent proteases were shown to play no role in DPC repair (Nakano et al., 2007). This same work demonstrated that small peptide crosslinks are efficiently removed by bacterial NER through its canonical mechanism, while bulky DPCs at stalled replication forks are substrate for HR-dependent damage tolerance, only to allow cell survival and replication in spite of the lesion that is left unrepaired. Later on, Salem and colleagues investigated the role of multiple *E. coli* DNA repair and translesion synthesis components on DPC tolerance *in vivo* (Salem *et al*., 2009). Their research confirmed that TLS and *Mfd*-mediated transcription coupled repair do not act on DNA-protein crosslinks, and replication restart is PriA-dependent, similarly to RecBCD-promoted HR. Also, Cho nuclease, rather than UvrC, seems to be responsible for the DNA incision downstream of the crosslinked peptide during NER. It is also possible that the UvrD helicase prevents unnecessary and deleterious HR by disassembling RecA nucleofilaments. *E. coli* exonuclease VII (XseA and XseB) was also implicated in the degradation of ssDNA ends next to DPCs (Liu et al., 2024), although the pathway comprising this complex has not been determined. 

## Conclusions

As addressed in this review, bacteria can be affected by crosslinking damage, being frequently exposed to agents produced by their own metabolism, other microbial and hosts, or other environmental sources. DNA crosslinks are highly cytotoxic and must be repaired, or at least bypassed, to ensure cell survival. Common DNA repair pathways such as BER and NER seem to be the major players for lesion recognition and removal, in concert with HR and TLS for ICL repair, although other systems also exist. Nonetheless, we believe that the small number of studies published on this subject, especially since working with eukaryotic cells became less challenging, points to an underexplored area where novel mechanisms can be discovered and characterized. 
